# Artificial Lighting as a Vector Attractant and Cause of Disease Diffusion

**DOI:** 10.1289/ehp.1002115

**Published:** 2010-08-01

**Authors:** Alessandro Barghini, Bruno A. S. de Medeiros

**Affiliations:** 1 Laboratório de Estudos Evolutivos Humanos, Departamento de Genética e Biologia Evolutiva, Instituto de Biociências, and Instituto de Eletrotécnica e Energia and; 2 Departamento de Zoologia, Instituto de Biociências, Universidade de São Paulo, São Paulo, Brasil

**Keywords:** Chagas disease, electricity, insect, leishmaniasis, lighting, malaria, vector

## Abstract

**Background:**

Traditionally, epidemiologists have considered electrification to be a positive factor. In fact, electrification and plumbing are typical initiatives that represent the integration of an isolated population into modern society, ensuring the control of pathogens and promoting public health. Nonetheless, electrification is always accompanied by night lighting that attracts insect vectors and changes people’s behavior. Although this may lead to new modes of infection and increased transmission of insect-borne diseases, epidemiologists rarely consider the role of night lighting in their surveys.

**Objective:**

We reviewed the epidemiological evidence concerning the role of lighting in the spread of vector-borne diseases to encourage other researchers to consider it in future studies.

**Discussion:**

We present three infectious vector-borne diseases—Chagas, leishmaniasis, and malaria—and discuss evidence that suggests that the use of artificial lighting results in behavioral changes among human populations and changes in the prevalence of vector species and in the modes of transmission.

**Conclusion:**

Despite a surprising lack of studies, existing evidence supports our hypothesis that artificial lighting leads to a higher risk of infection from vector-borne diseases. We believe that this is related not only to the simple attraction of traditional vectors to light sources but also to changes in the behavior of both humans and insects that result in new modes of disease transmission. Considering the ongoing expansion of night lighting in developing countries, additional research on this subject is urgently needed.

The expansion of nocturnal lighting has raised many concerns, the most prominent of these are the consumption of fossil fuels for electric power generation and the obstruction of views of the night sky and astronomical observation (Claudio 2009). At present, researchers have raised concerns that light pollution is also related to human health (Chepesiuk 2009; Holzman 2010; Stevens et al. 2007), mainly on the basis of chronobiological disorders. Calling attention to the fact that light pollution is also a major source of alterations to ecosystems, Longcore and Rich (2004) and Rich and Longcore (2006) coined the term “ecological light pollution.” By affecting the trophic web, light pollution can also, indirectly, influence human health.

Alternative energy sources such as solar photovoltaic panels and the new techniques applied in the production of artificial lighting, such as fluorescent bulbs and light-emitting diodes that are more efficient than traditional lighting (incandescent bulbs), will increase the accessibility of these resources to populations in developing countries that did not have access to them in the past (Mills 2004). The alternative forms of energy both will decrease the adverse effects of electricity on the environment and will allow the electrification of more isolated areas (International Energy Agency 2002). From a social perspective, these initiatives are beyond doubt positive. However, we should not forget that the areas around the world that lack electricity are mainly rural in equatorial and tropical regions where insect-transmitted diseases are prevalent (Jones et al. 2008). Because artificial illumination is a great attractor for insects, we believe that the diffusion of this electrification could constitute a risk of epidemic outbreaks of both existing and emerging diseases.

## Traditional Views on the Role of Electrification

Electrification is doubtless important as a means to develop rural areas, and it also has many beneficial effects. According to the Independent Evaluation Group of the World Bank (2008), for example, it operates through a number of channels: “improvements to health facilities; better health from cleaner air as households reduce use of polluting fuels for cooking, lighting, and heating; improved health knowledge through increased access to television; better nutrition from improved knowledge and storage facilities from refrigeration.” Most epidemiological reports cite both electrification and plumbing as positive factors in the control of diseases. As an example, Noor (2008) used “remotely sensed night-time light as a proxy for poverty in Africa,” indirectly assuming that artificial lighting is a good social development index. However, electrification also means artificial lighting, and artificial lighting is a strong insect attractant.

Although entomologists and epidemiologists traditionally have used light traps to capture insects, the effect on disease diffusion caused by the expansion of artificial lighting has generally not been considered—sometimes the effects have even been ignored. In fact, ignoring the effects of such light sources is long-standing: In the beginning of the 20th century when electrification started to diffuse in rural areas, epidemiologists did not consider its possible effects. For example, during the construction of the Panama Canal, Joseph Le Prince stated that it was man who attracted insects and that artificial lighting did not contribute to the diffusion of malaria (Le Prince and Orenstein 1916). Carlos Chagas, who discovered Chagas disease, noted that light is a good defense against the diffusion of the disease because *Triatoma*, its vector, does not bite in lighted areas (Chagas 1909).

Indeed, light may inhibit some insects from biting, but to understand its role in the diffusion of diseases, we need to take into account the behavioral changes in both human beings and insects. In other words, night lighting promotes new lifestyles, which, in turn, may lead to new modes of disease transmission.

Of course, we are not claiming that the introduction of modern lighting systems increases the risk of emerging diseases immediately and directly. The diffusion of electricity is bound to produce changes in human lifestyles, which are brought about by lighting, radio, television, and other electrical equipment. As a result of electrification, people may increase their outdoor activities in the evening; they may stay outdoors longer to exercise or to rest in hammocks or engage in other activities close to sources of bright light.

Light sources may increase people’s exposure to vectors merely by staying outdoors for longer periods of time in the evening, but vectors are also affected by light sources. In general, insects are attracted to lights. However, a common misunderstanding is that this attraction represents a positive phototaxis. Contrary to this belief, Thompson (1917), Verheijen (1958), Mazokhin-Porshnyakov (1969), Janzen (1983), and more recently Nowinszky (2003) observed that insect attraction to lights is the result of navigational confusion. Attraction results when insects mistake light sources (especially those emitting ultraviolet radiation) for the celestial points of reference they normally use for orientation, which may result in a trajectory toward a light. In the vicinity of a light source, however, not all insects are directly attracted to the lamp. Although some may be, others may hide in dark places around or near the light, fly in the illuminated area, or land somewhere near the lamp (Nowinszky 2008). Despite all this variation, it is important to stress that even vectors that usually bite only in the dark may be attracted to the surrounding areas of a light source and thus come into close contact to humans where they may transmit diseases in nonconventional ways. For example, Chagas disease was traditionally transmitted by triatomine bug bites, but oral transmission has become common with the advent of electrification, as we will discuss later.

To demonstrate the potential of night lighting for augmenting people’s exposure to vectors and for creating new modes of disease transmission, we performed a review of epidemiological studies that were conducted in rural equatorial regions. We found circumstantial evidence that electrification and lighting may be the source of new modes of transmission for three well-known infectious diseases.

## Chagas Disease

Ironically, the first confirmation of the strong impact of artificial lighting on the diffusion of diseases, validated by epidemiologists, came from Chagas disease. This is remarkable, considering that its vectors (triatomine bugs, also known as kissing bugs) do not bite in lighted areas, and artificial lighting has always been considered a good defense against them. Chagas disease was typically found among people living in adobe huts with straw-thatched roofs, excellent hideouts for the bug. It was widespread in pre-Columbian times in the Andean world, where domesticated cui or guinea pigs (*Cavia porcellus*) were the primary hosts (Coimbra 1988). The main vector was *Triatoma infestans*—a bug well adapted to households in which sanitary conditions are poor. In colonial times, it had spread to the South American lowlands, and by 1955–1964 the disease had reached central and northern Brazil, probably carried from place to place in the baggage of immigrants. The main vectors were *T. infestans* in Brazil, and *Rhodnius prolixus* in Venezuela, Colombia, and the Guyanas (Zeledon and Rabinovich 1981).

Large-scale insecticide spraying campaigns of households in Brazil after the 1970s and in most of Latin America after the 1980s proved to be effective in controlling Chagas disease in Brazil, but it continues to be a serious epidemic in Bolivia, Peru, Ecuador, and Venezuela. According to Dias et al. (2002),

By the end of the last century it became clear that continuous control in contiguous endemic areas could lead to the elimination of the most highly domestic vector populations—especially *Triatoma infestans* and *Rhodnius prolixus*—as well as substantial reductions of other widespread species such as [*Triatoma*] *brasiliensis*, [*Triatoma*] *sordida*, and [*Triatoma*] *dimidiata*, leading in turn to the interruption of disease transmission to rural people.

**Figure 1:**
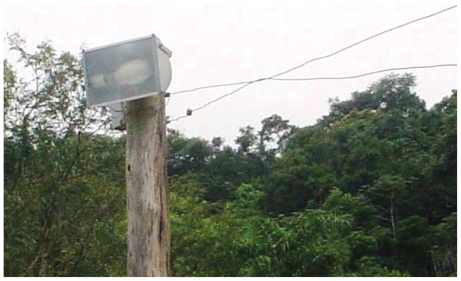
Triatomine bugs were attracted by this strong artificial light source at a sugarcane juice kiosk and were crushed together with the sugarcane when the juice was processed, which infected 12 people who consumed the juice. Photograph by L.A. Oliveira Ilha.

While eliminating the most highly domestic vector populations, new disease outbreaks arose, with a different pattern of diffusion involving a more diverse group of insect vectors and a larger pool of wild and domestic animal hosts. At the same time, a new mechanism of human transmission was discovered. Specifically, vectors that are attracted to artificial lighting in areas surrounding homes, instead of entering directly into homes. There, they may rest on plants such as the açai palm (*Euterpe oleracea*) and parasitize opossums or other warm-blooded animals. As a result, fruits contaminated with their feces may be consumed by people. This form of oral transmission is being increasingly observed and may be a consequence of the vector’s attraction to lighting. For example, in February–March 2005 the Department of Health of Santa Catarina (Brazil) identified an epidemic of Chagas disease (Ministério da Saúde 2007). After intensive research, it was verified that sugarcane juice sold at a roadside kiosk was the source of infection for all 12 confirmed cases. The vector of Chagas disease does not live in sugarcane plantations, and there was no reason for it to be in stored sugarcane. The only positive indication was the high-intensity discharge lamp installed at the sugarcane juice kiosk (Figure 1). The bugs (*Triatoma tibiamaculata*) were attracted by the strong artificial light source in the sugarcane juice kiosk and were crushed together with the sugarcane when the juice was processed, thus transmitting the infection.

The mechanism of oral transmission was originally proposed by Bertram (1971) and confirmed by Zeledon and Rabinovich (1981), who reviewed experiments on triatomine bugs and reported that 20 species—including *R. prolixus*—were attracted to lights. Many researchers have reiterated this hypothesis since then (Cuba et al. 2002; Feliciangeli et al. 2002; Salomon et al. 1999; Teixeira et al. 2001; Zeledon et al. 2001). In many cases reports simply mention the possibility that lighting may have facilitated disease transmission, but Walter et al. (2005) explicitly identified a strong association between the spread of Chagas disease and the use of kerosene lamps and photovoltaic panels. These are modern high-intensity lighting systems to which most insects are attracted.

Two recent important reviews of Chagas disease also concluded that artificial lighting may affect transmission of the disease (Remme et al. 2006; Rojas et al. 2005). Remme et al. (2006) described three different transmission cycles, including a domestic cycle involving domestic insect vectors and animal reservoirs that reside in close contact with humans, a sylvatic cycle in which sylvatic-insect vectors transmit the disease to wild animal hosts, and a peridomestic cycle in which sylvatic vectors that are attracted to lights in and around homes transmit infection by feeding on domestic animals and humans or indirectly transmit infection by contaminating food consumed by domestic animals and humans. In particular, they noted that in the Amazon region, humans have become infected with Chagas disease by eating sugarcane or fruit juice contaminated with the feces of sylvatic Triatominae.

## Leishmaniasis

Leishmaniasis is another disease whose spread appears to be augmented by artificial lighting. Sand flies (phlebotomines), the vectors of *Leishmania*, are poor flyers (Dias-Lima et al. 2002) that are attracted to lighted surroundings but are usually not found directly on lamps. In periurban areas, street lighting attracts sand flies to small farms or kitchen gardens, where dogs, chickens, and other small animals become the hosts. Campbell-Lendrum et al. (1999) showed that both *Lutzomyia intermedia* and *Lutzomyia whitmani* phlebotomine flies are attracted to light. Later, dos Santos et al. (2003) argued that this attraction may increase the risk of *Leishmania* transmission “in houses where an external light source is situated close to a light-color wall that reflects light, and that have adjacent bushes or trees and domestic animal shelters within 50 meters.”

Moreover, we cannot forget that sand flies are the vectors of a large number of arboviruses that are common in tropical and equatorial regions (Travassos da Rosa et al. 1998), which are the cause of a large number of diseases, generally called “wild fevers.” They are also vectors of infectious diseases in temperate regions, including West Nile encephalitis and equine encephalitis.

## Malaria

The case of malaria is more problematic. Unlike Chagas disease and leishmaniasis, there have as yet been no specific studies published on the relationship between night lighting and vector attraction. Although mosquitoes are seldom found near lamps and almost never captured by static light traps, they can be captured using suction light traps without heat or carbon dioxide bait (Govella et al. 2009; Jawara et al. 2008; Lee et al. 2009; Suárez-Mutis et al. 2009). Malaria vectors are therefore probably just as attracted to lights as are Chagas and leishmaniasis vectors, and we should then expect a corresponding change in modes of transmission with increased use of artificial light.

We also know that electrification is changing lifestyles in all isolated areas throughout the world. In Amazonia, for example, electric lights allow people to spend more time outdoors when vectors are active, particularly between sunset and the first hours of the night. Sports and gymnastics are practiced outdoors in the evening under strong artificial lights, and one may observe people resting in hammocks on their porches along the banks of the Amazon River, their electric lights shining brightly. These are all conditions that could affect vector attraction and also facilitate malaria transmission. However, we found no epidemiological studies on this matter relating to the Amazon.

Taylor (1997) proposed that increased time spent outdoors at night may have contributed to a resurgence in malaria infections among Solomon Island residents in the early 1980s, which followed substantial declines in infection rates resulting from in-home use of DDT (dichlorodiphenyltrichloroethane) in the 1960s. As Taylor (1975) observed, “Traditionally, the Melanesian peoples retired indoors at sunset but in more ‘enlightened’ areas this habit broke down (a combination of changed working hours and the money to buy artificial lighting).” Malaria control was regained in the Solomon Islands only ten years later when spraying was no longer limited to bed nets and households (Over et al. 2003). This suggests that night lighting augments human exposure to vectors by enabling people to stay outdoors longer. It is not clear if the vectors themselves were also attracted to lights or if lights affected their feeding behavior, but given that *Anopheles* are indeed attracted to light traps, these possibilities could be tested with additional research.

Other examples have come from two recent studies. Yamamoto et al. (2010), working in Burkina Faso, found that living in a home < 10 years old and in a home with electricity were both associated with an increased risk of malaria, whereas socioeconomic status was not a factor. The authors suggested that vectors might be more likely to bite residents of homes with electricity than residents of nonelectrified homes where greater use of biomass fuels would produce smoke that might prevent insects from biting. However, a recent review concluded that smoke does not reduce biting in homes (Biran et al. 2007). In South Africa, Coleman et al. (2010) found that opening windows at nighttime increases the risk of malaria transmission, but the authors did not evaluate electrification as an independent risk factor for disease transmission. In both studies, the researchers did not collect the necessary data to evaluate the role of artificial light.

## Conclusion

Although we have presented evidence that artificial light may increase the transmission of three diseases, we strongly believe that this is a consequence of a lack of studies rather than a lack of an effect and that the three diseases we have discussed may reflect a general pattern. Artificial night lighting changes the behavior of both people and insects and thereby promotes contact between human beings and vector species, including some that have not traditionally been involved in transmitting disease to humans. This may lead to new and unpredictable ecological relationships that need to be understood so that electrical energy can be offered to rural populations in areas where vector-borne diseases are endemic without increasing their risk of acquiring such diseases.

In order to properly test this hypothesis, the presence of night lighting in or near households must be recorded in epidemiological surveys, especially in recently electrified rural areas. We trust that this contribution will shed light on this neglected problem and encourage epidemiologists to carry out studies that take into account changes in human and vector behavior that is related to artificial lighting.
